# Influence of the anterior notch in mobile-bearing UKA on patellofemoral radiotracer uptake and clinical outcome

**DOI:** 10.1186/s12891-017-1885-6

**Published:** 2017-12-16

**Authors:** Dietmar Dammerer, Michael Liebensteiner, Hannes Rochau, Christian Uprimny, Vinzenz Smekal, Ralf Rosenberger, Elvire Servien

**Affiliations:** 1 Department of Orthopaedic Surgery, Medical University of Innsbruck, Anichstrasse 35, A - 6020 Innsbruck, Austria; 2 Department of Nuclear Medicine, Medical University of Innsbruck, Anichstrasse 35, A - 6020 Innsbruck, Austria; 3 AUVA Trauma Center Klagenfurt, Waidmannsdorf Straße 35, A – 9020, Klagenfurt, Austria; 4 Department of Traumatology, Medical University of Innsbruck, Anichstrasse 35, A - 6020 Innsbruck, Austria; 5 Department of Orthopaedic Surgery, Centre Albert-Trillat, Hôpital de la Croix-Rousse, 8 rue de Magnolles, 69300 Lyon, FR France

**Keywords:** Unicondylar knee arthroplasty, Unicompartmental knee arthroplasty, Patellofemoral, Notch, SPECT

## Abstract

**Background:**

Previous studies reported that in partial knee arthroplasty smooth transitions to the remaining native parts of the knee are important. However, in mobile-bearing unicondylar knee arthroplasty (UKA) it is mandatory to create an anterior osteochondral notch adjacent to the femoral component to get clearance for the anterior lip of the bearing in full knee extension. This notch is, however, part of the femoral trochlea.

It was the aim of the study to test for a potential association between a) an obligatory anterior notch in mobile-bearing UKA located at the margin of the medial aspect of the femoral trochlea and b) postoperative patellofemoral joint (PFJ) bone remodelling and discomfort.

**Methods:**

In patients who underwent routine mobile-bearing UKA (11 male, 13 female; 64.5 years / IQR 14) the following parameters were prospectively determined i) size of the surgically created anterior notch, ii) knee score sensitive to PFJ disorders, iii) bone remodelling in the PFJ (radiotracer uptake in SPECT-CT).

**Results:**

Notch size was not correlated with radiotracer uptake at the PFJ. Similarly, no significant correlations were observed between radiotracer uptake (patella or trochleocondylar junction) and knee scores (KOOS or Kujala Score). Significant *positive* correlations were found between notch size and knee scores.

**Conclusions:**

From the findings made in our study it is concluded that a larger size of the anterior notch in mobile-bearing medial Oxford UKA is not associated with increased osteochondral remodelling processes at the patella or the trochleocondylar junction. Neither is a larger sized notch associated with worse clinical PFJ outcome. Surprisingly, a larger notch was even associated with superior clinical outcome. The exact mechanism for this contraintuitive finding remains unclear but may be the basis for future research.

**Trial registration:**

The study is registered in a public trials registry. Link: (9/12/2017) ClinicalTrials.gov. NCT01407042; Date of registration: July, 26, 2011.

## Background

**Fig. 1:**
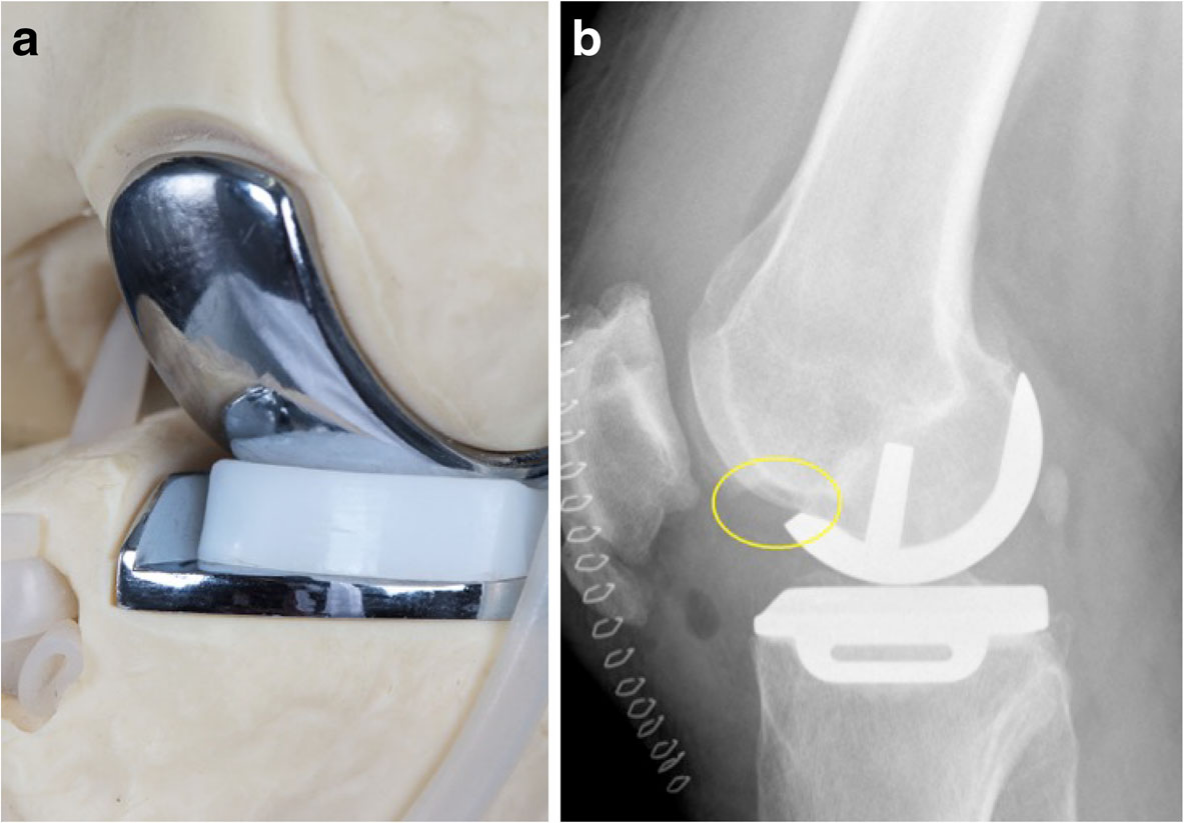
**a.** Saw bone model showing the anterior notch adjacent to the femoral implant (view from anteromedial). It is obligatory to create such a notch during mobile-bearing unicondylar knee arthroplasty **b.** Example of a rather large notch (10 mm) in one of our cases, as depicted in a lateral radiograph. The range of the notch size in our study population was 2–12 mm. The images were produced solely for this manuscript

In mobile-bearing unicondylar knee arthroplasty (UKA) creation of an osteochondral notch adjacent to the femoral implant is mandatory to prevent anterior impingement of the bearing (Fig. 1a). [1]. A notch size of 3 to 5 mm was recommended, but in daily practice the size necessary to prevent impingement in full extension can vary considerably, dependent on implant sizing, positioning and surgeon preference (Fig. 1b).

**Fig. 2:**
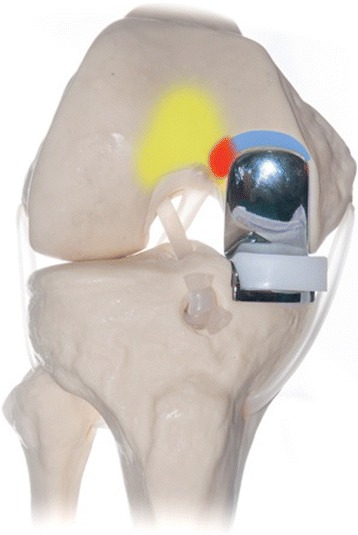
Overlap (red) between the notch required for the mobile-bearing UKA (blue) and the area where the patella articulates in deeper knee flexion (yellow). This region of overlap between the tibiofemoral and patellofemoral compartment is also known as trochleocondylar junction. (For illustration purposes only)

However, the lateral aspect of that notch is part of the deep femoral trochlea, where the patella articulates in deep knee flexion (Fig. 2). It was reported that even these ‚transition zones’ are exposed to relevant contact pressure [2]. Others emphasized the importance of a smooth transition between the femoral implant and the anterior region of the femur [3–5] (trochleocondylar junction). This is, however, not possible in mobile-bearing UKA as the surgeon has to create the above-mentioned notch to provide space for the anterior lip of the polyethylene insert.

It was the purpose of the study to investigate whether the surgically created, anterior notch in mobile-bearing UKA was associated with clinical PFJ outcome and PFJ radiotracer uptake. Consequently, we hypothesized that there would be significant correlations between the parameters 1) notch size, 2) clinical outcome in terms of PFJ sensitive knee scores and 3) PFJ radiotracer uptake.

## Methods

Patients scheduled for routine mobile-bearing UKA implantation due to osteoarthritis or osteonecrosis in the medial compartment were considered for enrollment. Contraindications were: 1) failed upper tibial osteotomy, 2) insufficiency of the collateral or anterior cruciate ligaments, 3) fixed varus or valgus deformity greater than 15°, 4) flexion deformity greater than 15°, and 5) rheumatoid arthritis. The study was conducted in accordance with the Declaration of Helsinki and informed consent was obtained from all subjects prior to participation.

All surgical procedures were performed under general or spinal anaesthesia under tourniquet control and after standard antibiotic prophylaxis. Patients received the ‘Oxford 3’ medial UKA (Zimmer Biomet Inc., Warsaw, Indiana, USA). The surgical technique was as recommended in the manufacturer’s surgical manual. Accordant to these recommendations an osteochondral resection was performed anterior to the implant to prevent impingement of the polyethylene inlay in full knee extension. After implantation the wound was closed over drainages. All operations were performed by an experienced consultant surgeon, and prophylaxis against venous thrombosis was administered in all cases. Patients underwent a standard rehabilitation program after surgery, consisting of continuous passive motion, and active and passive exercise under the guidance of a physiotherapist. Subsequently, most of the patients attended an outpatient rehabilitation program.

With regard to the above-mentioned hypotheses we determined the following outcome parameters: 1) notch size, 2) clinical PFJ outcome and 3) PFJ radiotracer uptake.

**Fig. 3:**
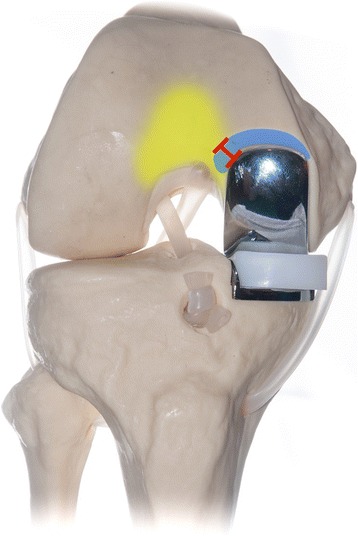
Notch size measurement is illustrated in red color. Notch size was determined at 10:30 o’clock in right knees and at 1:30 o’clock in left knees. This location was chosen to determine how far the notch extends in the direction of the deep femoral trochlea (where the patella articulates in deeper knee flexion)

**Fig. 4:**
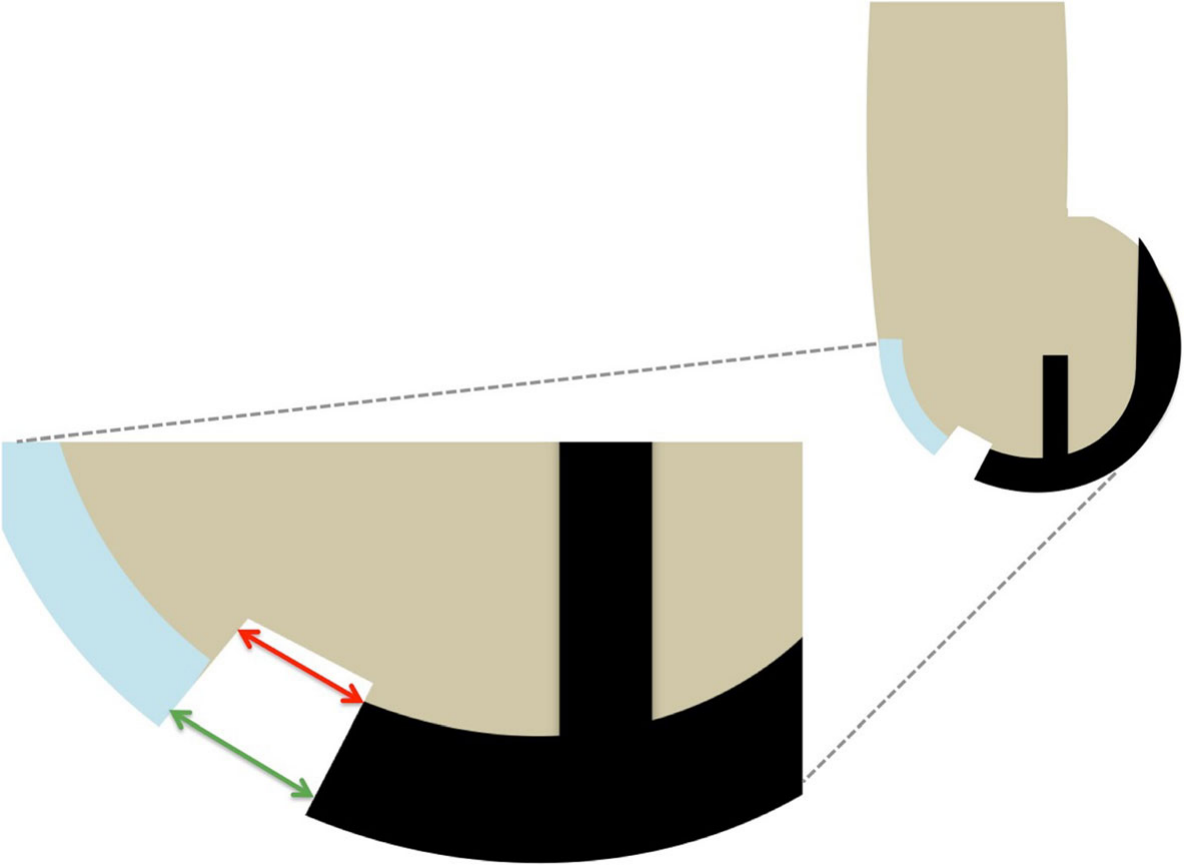
Notch size measurements were performed superficially at the cartilage level (green arrow) and at the bottom of the notch (red arrow)

The size of the notch adjacent to the femoral implant was measured intraoperatively by always the same observer using a sliding calliper. This was done at 10:30 o’clock in right knees and at 1:30 o’clock in left knees. This location was chosen to determine how far the notch extends in the direction of the deep femoral trochlea (where the patella articulates in deeper knee flexion) (Fig. 3). Measurements were done at the bony level (bottom of the notch) and at the cartilage level (surface of the notch) to determine the parameters ‘notch size bone’ and ‘notch size cartilage’, respectively (Fig. 4).

Clinical knee score outcome was assessed with the ‘Knee Injury and Osteoarthritis Outcome Score (KOOS)’ [6] and the Kujala Score [7]. High sensitivity for PFJ disorders, and favourable findings with regard to validity, reliability and responsiveness were reported for these scores [8–10]. On both the Kujala Score and the KOOS zero represents extreme knee problems, and 100 no knee problems. The knee scores were assessed preoperatively and six months postoperatively, always in a self-administered fashion.

PFJ bone remodelling around the notch was determined by ‘single photon emission computed tomography – computed tomography’ (SPECT-CT) six months after implantation. Three hours after administration of 500 MBq 99mTc-3,3-diphospho-1,2-propandicarbonacid (DPD; Teceos®) a whole body planar scan was acquired followed by a SPECT/CT of the knee region, covering a field of view of 45 cm. The exams were performed on a dedicated SPECT-CT system equipped with a dual-head gamma camera and a low-dose CT device (Philips Brightview XCT®). The particular value of SPECT-CT in assessing postoperative knee disorders was already demonstrated [11, 12].

**Fig. 5:**
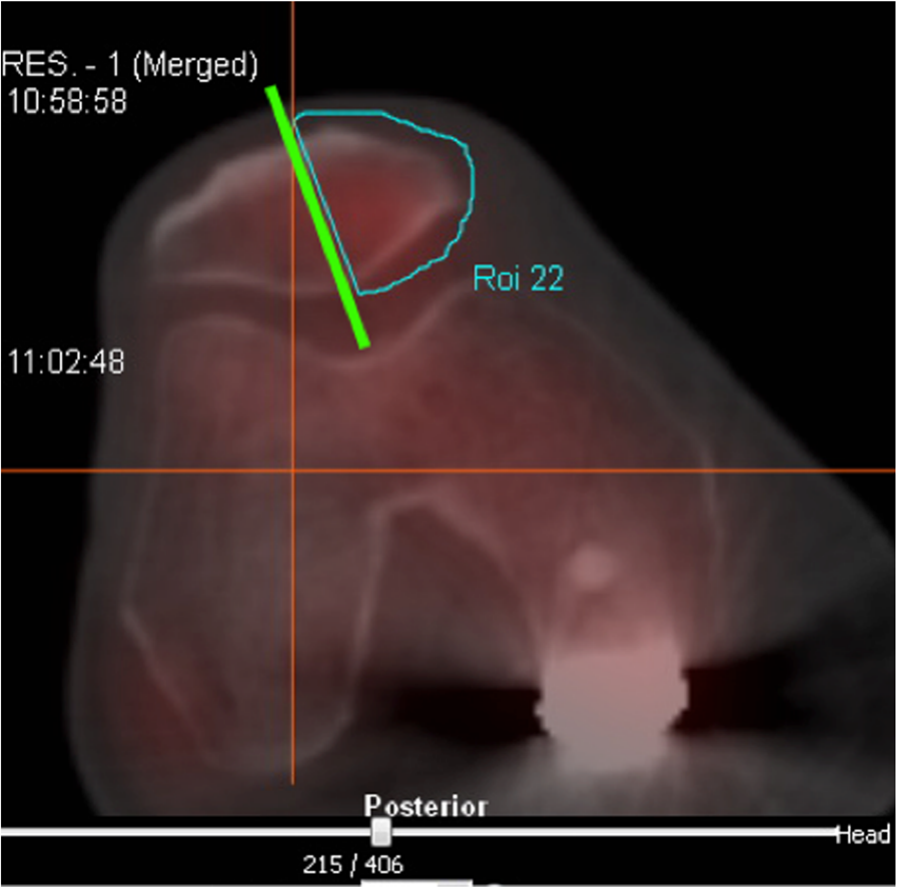
In transverse planes of the SPECT-CT fusion images regions of interests (ROIs) were defined manually. Here this process is shown for the medial facet of the patella. Many ROIs then comprised a volume of interest (VOI). Within the VOI the maximum tracer uptake was determined

Data processing was performed as follows. The Kujala Score and the KOOS were evaluated as described in the original publications [6, 7]. For analysis of SPECT-CT data the software ‘Hybrid Viewer’ (Hermes Medical Solutions, Stockholm, Sweden) was used. Two areas were of interest: the area of the notch and the entire medial facet of the patella. Around both areas volumes of interest (VOI) were determined with always the same standardized technique. We determined regions of interests (ROIs) in 2D manually e.g. in transverse slices of the medial patella. At the level of the patella’s maximum mediolateral width the mediolateral axis of the patealla was defined for reference purposes. A perpendicular line (anteroposterior direction) was defined and always positioned through the patellar ridge at the patella’s articular surface. A ROI was defined that included all aspects of the patella medial of that line (Fig. 5). Many ROIs then comprised the VOI. Within the VOI the maximum tracer uptake was determined. The maximum uptake was chosen as appropriate parameter a) because it was less dependent on variations in VOI definition and b) it was also chosen by previous studies dealing with SPECT-CT and knee disorders as appropriate outcome parameter [13–16].

Because of inter-individual tracer uptake differences caused by patient weight or variable tracer dispersion a ratio was calculated between the maximum uptake within the two above-mentioned VOIs and a reference VOI from cancellous bone from the contralateral femur (normalized maximum uptake).

For statistical analysis the software programme SPSS (International Business Machines Corporation, Armonk, NY, USA) was used. Because the case number was <30 the data were regarded as not normally distributed. Accordingly, medians and interquartile ranges (IQR) were determined as descriptives. For inferential statistics Spearman correlation coefficients were calculated and alpha was defined as 0.01 because of multiple analyses. Correlation strength was defined as follows: very high from 0.90 to 1.00, high from 0.70 to 0.90, moderate from 0.50 to 0.70, low from 0.30 to 0.50 and negligible from 0.00 to 0.30 [17]. The sample size was chosen pragmatically.

## Results

The 24 patients (11 male, 13 female) had a median age of 64.5 years (IQR 14) and a body mass index of 27.84 (IQR 3.99). 11 patients underwent right-sided UKA and 13 patients left-sided UKA. The patients’ mechanical tibiofemoral angle was 2.5° varus preoperative and 2.0° postoperative.

The KOOS and the Kujala Score improved from preoperative to 6 months postoperative (Table 1), but this was not linked to a hypothesis or inferential statistical tests. The notch size was 7 mm at the cartilage level (IQR 3.5, range 3–12 mm, 95%: 6.3–8.3) and 4.25 mm at the bone level (IQR 3.5, range 2–12 mm, 95% CI: 4.4–6.5).

**Table 1:** Descriptive statistics of the Kujala Score and the KOOS subscales from preoperative to 6 months postoperative (IQR: interquartile range; ADL: activities of daily living; QoL: quality of life)

		Median	IQR	95% CI
Kujala preoperative		46	19	41.9–51.7
Kujala 6mo		77	31	67.6–82.5
KOOS symptoms	preoperative	48	32	42.5–57.6
KOOS pain		36	13	33.4–44.8
KOOS ADL		40	23	36.3–49.6
KOOS sport		15	34	13.6–28.1
KOOS QoL		12	27	12.1–26.3
KOOS symptoms	6 months	79	33	66.0–82.2
KOOS pain		84	22	69.7–86.2
KOOS ADL		78	31	69.3–86.0
KOOS sport		60	39	46.5–68.3
KOOS QoL		59	39	50.8–70.3

With regard to our hypotheses the size of the anterior notch showed no significant correlations with the maximum tracer uptake at the patella or the trochleocondylar junction (p = n.s.). Similarly, no significant correlations were seen between the maximum tracer uptake (patella or trochleocondylar junction) and the knee scores (KOOS or Kujala Score) (p = n.s.). However, \several low and moderate *positive* correlations were found between the notch size and the knee scores 6 months postoperatively (see Table 2 for details).

**Table 2:** Correlation analysis between the notch sizes (cartilage level and bone level) and the knee scores at six months postoperative

			notch cartilage	95% CI	notch bone	95% CI
Kujala 6mo		r	0.458	0.067–0.727	0.440	0.045–0.752
		p	0.024	–	0.032	
KOOS symptoms	6 months	r	0.635	0.312–0.826	0.501	0.123–0.752
		p	0.001	–	0.013	–
KOOS pain		r	0.352	−0.059-0.661	0.345	−0.067-0.656
		p	0.092	–	0.099	–
KOOS ADL		r	0.501	0.123–0.752	0.375	−0.033-0.676
		p	0.013	–	0.071	–
KOOS sport		r	0.257	−0.163-0.598	0.391	−0.014-0.686
		p	0.226	–	0.059	–
KOOS QoL		r	0.564	0.208–0.788	0.585	0.238–0.799
		p	0.004	–	0.003	–

Significant correlations are marked in grey. (r: Spearman correlation coefficient; p: *p* value; ADL: activities of daily living; QoL: quality of life)

## Discussion

The most important findings were the *absence* of significant correlations between a) notch size and PFJ radiotracer uptake or b) knee score outcome and PFJ radiotracer uptake and the *presence* of significant correlations between c) ‚PFJ-sensitive’ knee scores and notch size.

Although the PFJ was already issued in the context of UKA in previous research [18–22], the specific issue of the current study has so far not been dealt with. No previous researchers applied SPECT-CT in a case series of mobile-bearing UKA to determine PFJ bone remodelling in the trochleocondylar junction. The findings of this study suggest that the size of the notch is not related to the amount of radiotracer uptake either at the notch itself or at the articulating part of the patella. Previous research suggested that increased radiotracer uptake can be interpreted as increased bone stress [23]. Therefore, it may be concluded that greater notch sizes are not associated with increased stress at the notch itself or the articulating aspects of the patella.

Low and moderate correlations were found between ‚PFJ-sensitive’ knee scores and notch size. Surprisingly, the coefficients indicated an inverse relationship to what had been assumed by us: the bigger the notch the better the knee score. Thus, based on the findings it can be concluded that a larger notch size in UKA does not negatively influence PFJ function. Indeed, it seems that a larger notch was associated with superior clinical outcome. It might be speculated whether, when in doubt, a larger anterior notch should be resected in medial mobile-bearing UKA. However, due to the small sample size our findings should be interpreted with caution.

Several authors reported on *fixed-*bearing UKA and emphasized the importance of a smooth transition between the anterior part of the femoral implant and the trochlea [3–5]. With regard to *mobile*-bearing UKA previous researchers reported anterior impingement of the bearing on the femur as a possible reason for surgical reintervention [24, 25]. We believe that this specific mechanism might also be applicable when interpreting the findings of the current study. This means that a larger notch might better prevent bearing impingement in full extension However, the prevention of anterior impingement of the bearing might not be the only potential reason for the positive correlations between knee scores and notch size. It might as well be speculated that notch size is just a surrogate variable for alignment of the femoral component in the sagittal plane. Implantation of the femoral component in slight flexion usually leads to more protuberance of the anterior lip of the bearing (over the anterior margin of the femoral component) in full knee extension. Consequently, a larger notch is created by the surgeon to accommodate for this protuberance and to prevent impingement of the bearing at the femoral bone. In other words, it could be that positioning the femoral component in slight flexion is the true factor that leads to superior score outcome. With this regard, it is also interesting that the manufacturer just recently recommended positioning the femoral component in slight flexion.

It is surprising that the notch size did not correlate with a biomechanically more closely related parameter, i.e. the radiotracer uptake, whereas it did correlate with a more high-level outcome parameter such as the knee scores. The exact reason for that counterintuitive constellation is not clear to us.

It is also surprising that the correlations between notch size and knee scores were observed heterogenously over type of subscores (KOOS symptoms and KOOS quality of life) (Tab. 2). We cannot provide explanations for the inhomogenous incidence of correlations over the different subscores.

Interestingly, the manufacturer of the Oxford mobile-bearing UKA recently introduced a special milling system to create the notch adjacent to the femur in a standardized fashion (Art.No. 32–423,238 and 32–423,239). Thus, the anterior notch in mobile-bearing UKA would appear to be a technical detail that is currently receiving increased attention.

The following limitations are acknowledged and at the same time may also be regarded as recommendations for future studies. First and foremost, as only medial UKAs were included in the study, the findings are of course valid only for *medial* mobile-bearing UKA. As the extensor apparatus relies more on the lateral trochlear facet, it can be speculated whether the same findings can also be determined for lateral mobile-bearing UKA. Second, it is regarded as limitation that we did not prospectively collect full-length radiographs to determine component alignment. For this reason it was not possible to test for relationships between component alignment and notch size. Third, we did not analyse radiographs of the PFJ of our patients over longer follow-up periods. We therefore cannot completely rule out the possibility that in the long run a larger notch provokes degenerative changes of the PFJ. However, the SPECT-CT revealed no corresponding increase in PFJ radiotracer uptake. In addition, the limited follow-up and a relative small study population is acknowledged. We could not get ethical approval for more patients due to the invasiveness of the SPECT-CT (high exposure to radiation). Moreover, it should be acknowledged that we did not perform an analysis of the repeatability and reliability of the notch-size measurements.

Nevertheless, the study at hand is the first publication to investigate the consequences of the requisite anterior notching in mobile-bearing UKA on the PFJ. We therefore believe it contributes to the current scientific knowledge. The strengths of the study also lie in the in-vivo measurement of PFJ bone remodelling with SPECT-CT fusion, as stimulated by previous publications [26, 27].

## Conclusions

From the findings made in our study it is concluded that a larger size of the anterior notch in mobile-bearing medial Oxford UKA is not associated with increased osteochondral remodelling processes at the patella or the trochleocondylar junction. Neither is a larger sized notch associated with worse clinical PFJ outcome. Surprisingly, a larger notch was associated with superior clinical outcome. The exact mechanism for this contraintuitive finding remains unclear but may be the basis for future research.

## Abbreviations

IQR: interquartile range
KOOS: knee injury and osteoarthritis outcome score
LEFS: lower extremity functional scale
n.s.: not significant
PFJ: patellofemoral joint
SPECT-CT: single-photon emission computed tomography – computed tomography
UKA: unicondylar knee arthroplasty
VOI: volumes of interest
